# New Drugs, Appliances, and Things Medical

**Published:** 1889-10-19

**Authors:** 


### NEW DRUGS, APPLIANCES AND THINGS MEDICAL.

### CHANCELLORS' BUDGETS AND CADBURY'S COCOA.

Well may chancellors, worried by budgets, shake their sad heads over a failing revenue. Stronger than financial forecast, the unwritten laws of fashion and of custom can fill or empty the coffers of a treasury with equal ease. Of late years a great change has come over the drinking habits of the nation, and the returns from excise have steadily diminished to the delight of the temperance party. But what is good news to the social reformer brings anything but unmixed joy to the Government that has to provide ways and means for working the machinery of national administration. The present head of the Exchequer, in bringing forward his budget a few years since, accounted for the falling off in excise partly by the greater advertising enterprise of the cocoa manufacturers. Although there is doubtless a great deal of truth in that statement, yet there are other important causes for the increased consumption of cocoa. Among them may be mentioned chiefly:

1. Disuse of alcohol.
2. Efforts of temperance reformers.
3. Cheaper non-alcoholic beverages.

Of these, the third may be looked upon as a supply for the demand created by the operation of the first two factors. It will be sufficient to remark, in passing, that the waning popularity of alcohol is likely to be no less characteristic of the present century than that other remarkable social change, the disuse of arms. Men are beginning to understand better the rules of right living, and to comprehend that happiness depends largely on personal health and unimpaired heredity. Alcohol has been repeatedly shown to be not only unnecessary, but harmful to the individual and to the race. Hence the crusade of the teetotallers may be regarded as a great and praiseworthy social mission. Apart from the overstatement common to enthusiasts, the cause of Sir Wilfrid Lawson must commend itself to the common-sense of the community and of all scientific men. Teetotalism is worthy of a foremost place among the preventive measures on which the future happiness of the "highest mammal" will largely depend.

As to the increase of non-alcoholic beverages, water is now plentiful and good, and there has been an enormous development in the mineral and aërated water trade. Tea and coffee have become very much cheaper, and many preparations of chocolate and cocoa are in the market.

Of the latter we have lately been favoured with samples of an essence made by our old friends Messrs. Cadbury Brothers, of Birmingham. As most people know, genuine cocoa contains a considerable amount—as much as 50 per cent.—of fatty matter, or "butter," which makes ordinary cocoa indigestible for many people. In some of the Dutch and home cocoas this excess of fat is counteracted by the addition of starch, arrowroot, alkali, and other agents. Messrs. Cadbury, however, adopt a more scientific means of meeting the difficulty, namely, by extracting about two-thirds of the fatty matter from the powdered bean. Their mode of preparation leaves a perfectly soluble essence, of fine aroma and easy of digestion. There can be nothing better than a well-made cup of cocoa, and many people would be wise to drink it instead of afternoon tea. There can be little doubt that tea—at any rate as we now drink it—has much to answer for in the frequency of latter-day dyspepsia, due to chemical qualities from which cocoa is entirely free. Tea is an old and valued friend, but there is good reason to recommend the substitution of beverages less likely to do harm, and on this score we advise all who are threatened with stomachic woes to give cocoa a trial.

